# EXTUBATE: A randomised controlled trial of nasal biphasic positive airway pressure vs. nasal continuous positive airway pressure following extubation in infants less than 30 weeks' gestation: study protocol for a randomised controlled trial

**DOI:** 10.1186/1745-6215-12-257

**Published:** 2011-12-09

**Authors:** Suresh Victor

**Affiliations:** 1 Ward 68, 2nd Floor, St Mary's Hospital for Women and Children, Manchester, UK M13 9WL

**Keywords:** Preterm birth Infant, Newborn, Humans, Clinical trials, Randomized, Neonatal Intensive Care, Biphasic Continuous Positive Airway Pressure, Continuous Positive Airway Pressure

## Abstract

**Background:**

Respiratory distress syndrome remains a significant problem among premature infants. Mechanical ventilation through an endotracheal tube remains the mainstay of respiratory support but may be associated with lung injury and the development of chronic lung disease of prematurity. Efforts are needed to reduce the duration of mechanical ventilation in favour of less invasive forms of respiratory support and to improve rates of successful extubation.

Non-invasive respiratory support has been demonstrated to be less injurious to the premature lung. Standard practice is to use nasal continuous positive airway pressure (n-CPAP) following extubation to support the baby's breathing. Many clinicians also use nasal biphasic positive airway pressure (n-BiPAP) in efforts to improve rates of successful extubation. However, there is currently no evidence that this confers any advantage over conventional nasal continuous positive airway pressure.

**Methods:**

We propose an unblinded multi-centre randomised trial comparing n-CPAP with n-BiPAP in babies born before 30 weeks' gestation and less than two weeks old. Babies with congenital abnormalities and severe intra-ventricular haemorrhage will be excluded. 540 babies admitted to neonatal centres in England will be randomised at the time of first extubation attempt. The primary aim of this study is to compare the rate of extubation failure within 48 hours following the first attempt at extubation. The secondary aims are to compare the effect of n-BiPAP and n-CPAP on the following outcomes:

1. Maintenance of successful extubation for 7 days post extubation

2. Oxygen requirement at 28 days of age and at 36 weeks' corrected gestational age

3. Total days on ventilator, n-CPAP/n-BiPAP

4. Number of ventilator days following first extubation attempt

5. pH and partial pressure of carbon dioxide in the first post extubation blood gas

6. Duration of hospital stay

7. Rate of abdominal distension requiring cessation of feeds

8. Rate of apnoea and bradycardia

9. The age at transfer back to referral centre in days

The trial will determine whether n-BiPAP is safe and superior to n-CPAP in preventing extubation failure in babies born before 30 weeks' gestation and less than two weeks old.

**Trial registration number:**

ISRCTN: ISRCTN18921778

## Background

Our working hypothesis is that preterm infants born before 30 weeks' gestation and less than two weeks old when extubated on to nasal biphasic positive airway pressure (n-BiPAP) will have a lower risk of extubation failure than infants extubated on to single level, nasal continuous positive airway pressure (n-CPAP).

Respiratory distress syndrome due to surfactant deficiency is almost invariable in infants born at less than 28 weeks' gestation and remains a significant problem in infants born up to 34 weeks' gestation. Mechanical ventilation is the mainstay of treatment in this condition and may be required for prolonged periods of time extending to several weeks. Mechanical ventilation through an endotracheal tube is associated with ventilator associated lung injury which may be a significant factor in the development of chronic lung disease of prematurity (Bronchopulmonary dysplasia) - a condition associated with significant morbidity and mortality among preterm infants. Efforts have been made to reduce dependence on mechanical ventilation using less invasive forms of respiratory support such as nasal continuous positive airway pressure (n-CPAP). In this study we aim to determine if the use of nasal biphasic positive airway pressure (n-BiPAP) is more effective than single level variable flow n-CPAP in preventing extubation failure in babies born before 30 weeks' gestation and less than 2 weeks old.

n-CPAP has previously been shown to reduce the risk of extubation failure among preterm infants following extubation from mechanical ventilation. However, in around 25% of infants attempts at extubation fail [1]. Non-invasive respiratory support has been demonstrated to be less injurious to the preterm lung [2]. Efforts directed to minimise the exposure to or duration of mechanical ventilation in preterm infants have demonstrated the feasibility of early n-CPAP support among extremely preterm infants [3]. In this study, we hypothesise that there can be a further 10% reduction in extubation failures using n-BiPAP compared with n-CPAP.

Nasal Intermittent Positive Pressure Ventilation (NIPPV) has been used as respiratory support post extubation and there is evidence supporting the superiority of NIPPV over conventional n-CPAP in avoiding re-intubation in preterm infants [4]. Infants with birth weight less than 2000 g and mild surfactant deficient lung disease demonstrated reduced work of breathing with synchronised NIPPV compared with conventional n-CPAP [5]. Both of these studies involved the use of the Infant Star ventilator providing continuous flow CPAP with a Star Synch abdominal capsule to deliver synchronised NIPPV. The provision of synchronised NIPPV requires a ventilator capable of delivering this mode of support. The Infant Flow Advance offers a less complex/invasive method for the delivery of n-CPAP/n-BiPAP. n-CPAP with the Infant Flow Driver (a variable flow device) has also been shown to be more effective than CPAP delivered using a continuous flow device in improving oxygenation and reducing work of breathing (see below).

The Infant Flow Advance delivers positive pressure at the nose at two set pressure levels or n-BiPAP. The upper pressure setting is set 3 to 5 cm of water above the base pressure and is delivered for a set "inspiratory" time and a set "rate". With n-BiPAP, there is no synchronisation with the infant's respiratory effort; the infant continues to breathe spontaneously throughout the periods of baseline and upper pressure delivery. There have been two published trials to date which describes the use of n-BiPAP in preterm infants [6,7]. Migliori et al performed an unblinded crossover study comparing four alternating phases of n-CPAP and n-BiPAP in twenty infants (gestational ages 24 to 31 weeks) within 6 hours of weaning from mechanical ventilation. Significant improvements in oxygen saturations and transcutaneous partial pressure of oxygen and carbon dioxide were noted during the n-BiPAP phases. There was also a significant reduction in spontaneous respiratory rate during the n-BiPAP phases. Lista et al conducted a randomised control trial of 40 babies comparing the use of n-BiPAP with n-CPAP in premature infants following Intubation-Surfactant-Extubation (InSurE) approach [7]. They showed a significant reduction in duration of respiratory support, duration of oxygen dependency and gestational age at discharge in the group receiving n-BiPAP [7].

The most commonly used method for providing n-CPAP in the UK is the Infant Flow Driver, which provides variable flow CPAP support. The Infant Flow Driver is a device which generates positive pressure at the nose by the action of a jet of gas that changes direction with variations in flow/pressure within the nose itself, reflecting the infant's respiratory activity [8]. In this way, there is variable flow, according to the phase of the infant's spontaneous respiratory effort. Prior evidence has demonstrated that n-CPAP delivered using an Infant Flow Driver is more effective both at recruiting lung volume and reducing the work of breathing in preterm infants compared with continuous flow devices [9,10]. The Infant Star ventilator delivers continuous flow n-CPAP, so the published comparisons between n-CPAP and synchronised NIPPV cited above may not be directly applicable to variable flow n-BiPAP provided using the Infant Flow Advance. The Infant Flow Advance delivers bi-level pressure by increasing the flow into the inspiratory limb of the circuit by up to 5 litres/minute (depending on the desired pressure). This increased flow enters the circuit proximal to the humidifier and there is an intrinsic time lag in delivering the increased pressure (the rise time is at best " < 1 second" - internal communication from Viasys Healthcare), which poses some doubt over whether effective synchronisation with the infant's inspiratory effort is feasible and whether the additional complication of an abdominal capsule to facilitate attempts at synchronisation is necessary.

We therefore seek to compare the use of n-BiPAP with conventional variable flow n-CPAP using the Infant Flow Advance for respiratory support post extubation in preterm infants born before 30 weeks' gestation.

### Relevance of the study to patients and service providers

The proposed study will be the first randomised trial that will aim to compare the effectiveness of n-BiPAP vs. n-CPAP in preventing extubation failure in premature babies born before 30 weeks' gestation and less than two weeks old. It will allow clinicians to have an evidence based approach to the use of n-BiPAP under these circumstances. Currently, the device is often used with shorter 'inspiratory times' which significantly compromises its performance (S Mitchell, personal communication) and without good evidence to support either its benefit or its safety.

If our hypothesis is proved correct and the use of n-BiPAP improves the chances of successful extubation, then the regular use of n-BiPAP would reduce the number of ventilator days in preterm infants. Reducing the number of ventilator days is one way of minimising chronic lung injury in premature infants. Earlier weaning and hence the reduction in number of ventilator days could have a significant positive impact on neonatal services & resources.

In the UK, facilities for the mechanical ventilation of extremely premature infants tend to be concentrated at neonatal tertiary referral centres in the National Health Service. Once infants are successfully managed off the ventilator they are transferred back to hospitals closer to home. Reduction in ventilator days would allow earlier transfer back to local hospitals with benefits to neonatal service provision and to families.

In England, 7000 preterm infants are born each year at high risk of developing chronic lung disease of prematurity/bronchopulmonary dysplasia. This condition is associated with significant morbidity and mortality. About half of affected infants continue to need supplemental oxygen at 36 weeks' gestation. The duration of ventilation during the newborn period is one of the aetiological factors involved in the development of this condition. Reduction in extubation failures and number of days on the mechanical ventilator may minimise ventilator associated lung injury and reduce this contribution to the development of chronic lung disease of prematurity. By using the Infant Flow Advance, this study is comparing two methods of delivering non-invasive support using the same widely available device in this group of infants.

## Methods

### Aims

The primary aim of this study is to compare the risk of extubation failures for 48 hours after the first extubation comparing the use of n-BiPAP or n-CPAP in infants born before 30 weeks' gestation and less than two weeks old.

### Study design

This will be an unblinded multi-centre randomised trial of n-BIPAP vs. n-CPAP in infants born before 30 weeks' gestation and less than two weeks old.

### Inclusion criteria

Infants satisfying the following inclusion criteria will be eligible to participate:

(1) Born before 30 weeks' gestation (and)

(2) Ventilated through an endotracheal tube (and)

(3) Less than two weeks old (and)

(4) Has not had first attempt at extubation (and)

(5) Parental consent has been obtained

### Exclusion criteria

Infants with the following exclusion criteria will be not be eligible to participate:

(1) Presence of major congenital malformations

(2) Presence of neuromuscular disease

(3) Presence of known upper respiratory tract abnormalities

(4) Infants requiring surgery and likely to be within 7 days post-operative at the point of extubation.

(5) Presence of intraventricular haemorrhage with parenchymal extension.

### Primary outcome measure

Failure of extubation during the first 48 hours post-extubation will be used as the primary outcome measure. 48 hours will be calculated from the hour of extubation. Failure of extubation has been defined later under 'Criteria for failure of extubation'. We have chosen 'Failure of extubation' as our primary outcome measure to maintain similarity with previous trials conducted using NIPPV. We will also measure the following secondary outcome measures.

### Secondary outcome measures

(1) Maintenance of successful extubation for 7 days from the hour of extubation

(2) Total days on ventilator, n-CPAP/n-BiPAP (anytime requiring respiratory support during a 24 hour clock will be counted as a day).

(3) Number of ventilator days following first extubation attempt (anytime requiring respiratory support during a 24 hour clock will be counted as a day).

(4) Oxygen requirement at 28 days of age and at 36 weeks' corrected gestation

(5) pH, partial pressure of carbon dioxide in the first post extubation gas

(6) Duration of hospitalisation

(7) Rates of abdominal distension requiring cessation of feeds for 7 days post extubation

(8) Rate of apnoea and bradycardia expressed as events per hour during the 48 hours following extubation

(9) Age at transfer back to referral centre in days

(10) Adverse events as described later

### Sample size

A sample size of 270 in each group will have 80% power to detect a 10% reduction in the rates of extubation failure from 25% in the n-CPAP group to 15% in the n-BiPAP group at a 0.05 two-sided significance level. A 10% reduction in extubation failure rate would be clinically significant and could support a change in practice to using n-BiPAP as first line treatment post-extubation. Existing meta-analyses of trials comparing NIPPV and n-CPAP had around 80 babies in each arm.

### Recruitment

Some preliminary written and oral information will, whenever possible, be offered to parents prior to birth if the baby is likely to be eligible. Additional information will be given once the baby has been born. Informed written consent will be sought from a parent only after they have been given a full oral and written explanation of the study.

Sufficient time will be provided for consent. In most circumstances it will be possible to provide 24 hours time for parents to make their decision. Occasionally, babies may be ready to come off the ventilator quickly. Under those circumstances less time may be available for parents to decide. Since both treatments are used in routine care we anticipate that this will not place parents under undue pressure.

Where there is sufficient time between recruitment and randomisation, parents will be offered an early appointment with the Principal Investigator or delegated deputy who will meet with them to ensure that they understand the trial procedures and continue to consent to participate in the trial. At all stages it will be made clear to the parents that they remain free to withdraw their baby from the study at any time.

Parents who do not speak English will only be approached if an adult interpreter is available. Trust interpreter and link worker services will be used to support involvement of participants whose first language is not English.

A senior investigator will be available at all times to discuss concerns raised by parents or clinicians during the course of the trial. Mrs Huma Aziz, Service User representative will also be available to talk to parents who require further information from another parent who has experience in participating in research.

Information about the study will continue to be offered to parents after their infant leaves the unit. A newsletter about the study will be disseminated at regular intervals.

### Randomisation

Infants will be randomised following the decision to extubate. Block randomisation will be used, stratified by centre and gestation (< 28 weeks or ≥ 28 weeks). Web based randomisation will be used.

### Blinding

The n-BiPAP device produces an audible noise which cannot be masked. Due to this, parents, clinicians involved in patient care and researchers assessing study end-points will not be blinded to the nature of the study treatments. To reduce bias, criteria for extubation and re-intubation have been defined and will be strictly adhered to.

### Minimisation of bias

This is an un-blinded randomised controlled trial. To minimise bias 'Criteria for extubation' and 'Criteria for failure of extubation' have been carefully defined. These will be strictly adhered to throughout the trial. Randomisation will be conducted once the decision to extubate has been taken. The time duration between randomisation and extubation will be closely monitored and explanations sought where there is undue delay. Auditing will also be done on adherence to 'Criteria for extubation' and 'Criteria for failure of extubation' during site visits.

### Criteria for extubation

These will be:

(1) Loaded with caffeine according to standard local protocol (and)

(2) Satisfactory blood gases defined as pH of more than 7.25 and partial pressure of carbon dioxide of less than 7 kPa (52.5 mmHg). As most babies will not have arterial access at the time of extubation we have not defined partial pressure of oxygen in targeted blood gas indices (and)

(3) Mean airway pressure of 7 cm water or less (and)

(4) Fractional inspired oxygen concentration of 35% or less (and)

(5) Good spontaneous respiratory effort, persistently higher than ventilator rate.

### n-CPAP

The n-CPAP group will receive at extubation a single level continuous positive airway pressure of 6 cm water for at least 48 hours before weaning is commenced. If the infant is stable for the preceding 24 hours defined by having fewer than three minor apnoeas and no increase in oxygen requirement, weaning will be permitted. Minor apnoea will be defined as apnoea requiring stimulation but not mask ventilation. CPAP will be decreased from 6 cm water by 1 cm water every 48 hours if tolerated based on the above criteria. This will be done until a pressure of 4 cm water is reached. If a pressure of 4 cm water is successfully tolerated for 48 hours then time off n-CPAP will be allowed. Thereafter, no fixed weaning regime based on number of hours in a day the infant will be allowed to come off CPAP will be prescribed.

### n-BiPAP

The n-BiPAP group will receive at extubation a mean airway pressure of 6 cm water (positive end expiratory pressure of 4 cm water and peak inspiratory pressure of 8 cm of water). Inspiratory time of one second and respiratory rate of 30/min will always be maintained. If the infant is stable for the preceding 48 hours at mean airway pressure of 6 cm water defined by having fewer than 3 minor apnoeas and no increase in oxygen requirement, weaning will be permitted. Minor apnoea will be defined as apnoea requiring stimulation but not mask ventilation.

The infant will then receive a mean airway pressure of 5 cm water (positive end expiratory pressure of 4 cm water and peak inspiratory pressure of 6 cm of water). If the infant is stable for the preceding 48 hours at mean airway pressure of 5 cm water defined by having fewer than 3 minor apnoeas and no increase in oxygen requirement, weaning will be permitted. Minor apnoea will be defined as apnoea requiring stimulation but not mask ventilation.

The infant will then receive a CPAP of 4 cm water. If the infant is stable for the preceding 48 hours at CPAP of 4 cm water defined by having fewer than 3 minor apnoeas and no increase in oxygen requirement, then time of CPAP will be permitted. Minor apnoea will be defined as apnoea requiring stimulation but not mask ventilation. Thereafter, no fixed weaning regime based on number of hours in a day the infant will be allowed to come off CPAP will be prescribed.

### Standards of care

Routine hourly monitoring of heart rate, peripheral oxygen saturation and respiratory rate will be performed. Delivered n-CPAP and n-BiPAP pressures will be monitored regularly. Efforts will be made to maintain desired CPAP and mean airway pressure levels in accordance with standard nursing practice.

### Criteria for failure of extubation

This will be defined as:

(1) Uncompensated respiratory acidosis defined as pH less than 7.2 and partial pressure of carbon dioxide of more than 8 kPa (60 mmHg) (or)

(2) Major apnoea requiring mask ventilation

### Rescue treatment

Rescue treatment with n-BiPAP will not be allowed. Mechanical ventilation through endotracheal tube will be required when 'Criteria for failure of extubation' are reached.

### Data collection

All data for trial analysis are routine clinical items that can be obtained from the clinical notes. Data will be collected on trial-specific case report forms. Data will be entered by the research nurse or Local principal investigator on a web based electronic case record form provided by OpenCDMS. Access to the form will be password protected and participants will be identified by trial number only.

Clinical information will be collected at the following times:

1. At trial entry: Information on eligibility; background information and randomisation

2. Following randomisation: Daily observations regarding ventilation, vital signs, blood gases, haemoglobin and C-reactive protein, apnoeas and de-saturations for 1 week after extubation

3. Follow-up: Data on duration of hospital stay, duration of oxygen requirement, oxygen requirement at 28 days and at 36 weeks corrected gestational age and age at transfer back to referral centre, total number of days on ventilator, n-CPAP and n-BiPAP

Further information will be collected on expected serious adverse events. No additional blood samples are required for this study.

### Statistical analysis

A formal statistical analysis plan and other covariates will be pre-specified after a blinded review of the data. The primary efficacy analysis will be conducted on an intention to treat basis. The primary outcome variable is a binary (yes/no) outcome variable and groups will be compared using a logistic regression model adjusting for the stratification variables and other covariates prior to the review stage Other outcomes will be analysed using analogous ordinary, ordinal or logistic regression models. Any formal sub-group analyses will be pre-planned and based on specific interaction tests.

### Interim analysis and termination of trial

No interim analyses are planned. If any such are advised by the Data Monitoring Committee (DMC) an analysis plan will be developed and agreed by the DMC and Trial Steering Committee (TSC). Haybittle-Peto stopping rules will be used.

### Duration of study

540 infants will be recruited over 2.5 years. The trial will terminate when the last recruited infant is discharged from hospital or dies.

### Quality control and quality assurance procedures

#### Compliance to protocol

Compliance will be defined as full adherence to protocol. Compliance with the protocol will be ensured by a number of procedures as described below.

#### Site set-up and training

Start-up visits at each site, including training in trial procedures, will be performed before the first infant is enrolled.

Regular site visits will be made by the Trial co-ordinator and/or the Chief Investigator or a delegated member of the Trial Management Group to ensure adherence to the protocol and to deal with any site specific issues. A major focus of these visits will be the quality of data collection, adherence to protocol and minimisation of bias.

Nurse study days will be undertaken to ensure that nurses involved with the study are fully appraised of issues such as consent, data collection issues and study specific procedures.

#### Data processing and monitoring

All study data will be

**1**. Screened for out-of-range data, with cross-checks for conflicting data within and between data collection forms by a data manager.

**2**. Referred back to relevant centre for clarification in the event of missing items or uncertainty.

**3**. A random 10% of the data will be independently validated against the source documents by the data manager.

**4**. The Chief Investigator and trial statistician will review the results generated for logic and for patterns or problems. Outlier data will be investigated. The Chief Investigator and Trial Statistician will decide if any action needs to be taken.

### Safety Definitions

#### Adverse event (AE)

Any untoward medical occurrence in a subject recruited to a clinical trial, including occurrences which are not necessarily caused by or related with the treatment. An adverse event can be any unfavourable and unintended sign or symptom associated with the use of the intervention whether or not related to its use.

#### Serious adverse event (SAE)

Any adverse event that

a) Results in death

b) Is life-threatening

c) Requires hospitalisation or prolongation of existing hospitalisation

d) Results in persistent or significant disability or incapacity

#### Expected serious adverse events

Due to the high risk population of extremely premature infants with gestational age under 30 weeks, the following are serious adverse events which could be expected for this population during the course of the study:

(1) Intraventricular haemorrhage defined as haemorrhage causing ventricular dilatation with or without brain parenchymal involvement

(2) Periventricular leukomalacia on cranial ultrasound scan imaging

(3) Necrotising enterocolitis requiring surgery

(4) Patent ductus arteriosus requiring treatment

(5) Retinopathy of prematurity requiring laser treatment

(6) Pneumothorax within 7 days after extubation

(7) Evidence of traumatic nasal injury

(8) Death

#### Unexpected serious adverse events

These would be any serious adverse events that were not expected to occur in this high risk population as listed above.

### Reporting of serious adverse events

All expected Serious Adverse Events will be recorded. All expected serious adverse events whether or not they are attributable to the study intervention will be reviewed by the local principal investigator to determine if there is reasonable suspected causal relationship to the intervention. If there is evidence that there may be a novel causal relationship with the intervention, then the procedure for expedited reporting of serious adverse events will be followed. All adverse events will be followed until satisfactory resolution or until the investigator responsible for the care of the participant deems the event to be chronic or the patient to be stable.

The DMC will receive an analysis of adverse events (grouped by body system, severity and randomised group) and overall safety data as part of the closed report at its 6 monthly meeting. The DMC will review the analysis and give recommendations to the TSC when appropriate.

All unexpected serious adverse events whether or not they are attributable to the study intervention will be reviewed by the local principal investigator to determine if there is reasonable suspected causal relationship to the intervention. The Trial Management Group (TMG) will regularly review unexpected SAEs reported and send the forms monthly to the sponsor, except where a causal relationship is suspected and expedited reporting procedures are necessary.

### Reporting of Serious Adverse Events with a suspected causal relationship to the intervention

All Serious Adverse Events with a suspected causal relationship to the intervention will be reported to the Chief Investigator within one working day of the discovery or notification of the event.

The Chief Investigator will report all these events to the Ethics Committee and Sponsor. Fatal or life threatening SAEs with a suspected causal relationship will be reported within 7 days and all other SAEs at monthly intervals after TMG review. In addition a copy of the SAE with a suspected causal relationship to the intervention will be forwarded to the Chair of the Data Monitoring Committee. The Chair will also be provided with a document detailing all previous SAEs with their allocation. The Chief Investigator will also inform all investigators concerned of relevant information about SAEs that could adversely affect the safety of participants.

All SAEs with a suspected causal relationship to the intervention that result in a participant's withdrawal from the study or are present at the end of the study will be followed up until a satisfactory resolution occurs.

### Trial governance

#### Trial Management Group (TMG)

This group including the user-representative will meet bi-monthly to review trial safety and recruitment.

#### Trial Steering Committee (TSC)

A TSC will be set up which will have overall supervision of the trial. It will meet prior to commencement of the trial and then 6 monthly until completion. A meeting of the TSC will be held within a month of every DMC meeting to consider their recommendations.

Membership of the TSC will include members of the TMG and Local Principal Investigators at recruiting sites. Additional independent members in the committee will also include a representative from the Sponsor, a representative from the NIHR network and the local Bliss Charity representative. The chair of the TSC will be an independent member. Voting rights will be such that there is equal representation from independent and non-independent members.

#### Data Monitoring Committee (DMC)

An independent DMC will be formed and constituted according to the DAMOCLES guidelines. This will review safety data, recruitment, and event rates (sample size assumptions). The DMC will recommend if any interim analysis of the study outcomes is necessary and agree the timing and content of any such analyses. A DMC Charter will be drawn up prior to the start of the trial taking account of the DAMOCLES statement.

The DMC will report to the TMG and TSC. The TMG will make decisions about the conduct of the study and inform the TSC of significant issues. The trial will be monitored in line with International Conference on Harmonisation- Good Clinical Practice by the Research and Development department at Central Manchester University Hospitals NHS Foundation Trust.

#### Research parent group (RPG)

The research parent group will meet around the same time as Trial Steering Committee. The research team will update them regarding progress since the last meeting including, rate of recruitment, any adverse events recorded and other trial related issues. The group will then be allowed to discuss the project independently with no researchers present. Following the discussion, the group will be given an opportunity to provide their feedback.

## Trial status

Ethics Committee approval was granted on 4^th ^January 2011. Recruitment has commenced on 1^st ^July 2011.

## List of abbreviations used

n-CPAP: Nasal Continuous Positive Airway Pressure; n-BiPAP: Nasal biphasic positive airway pressure; NIPPV: Non Invasive Positive Pressure Ventilation; DMC: Data Monitoring Committee; TSC: Trial Steering Committee; TMG: Trial Management Committee; RPG: Research Parent Group; AE: Adverse Event; SAE: Serious Adverse Event

## Competing interests

The author declares that they have no competing interests.

## Authors' contributions

SV, SM, HA, SR, TL have contributed to (1) the conception and design of the trial; (2) have been involved in drafting the manuscript; (3) have given final approval of the version to be published.

This paper presents independent research funded by the National Institute for Health Research (NIHR) under its Research for Patient Benefit (RfPB) Programme (Grant Reference Number PB-PG-0909-19171). The views expressed are those of the author(s) and not necessarily those of the NHS, the NIHR or the Department of Health.

## Author's information

Suresh Victor, Clinical Lecturer and Honorary Consultant Neonatologist, Newborn Intensive Care Unit, Central Mcr University Hospitals NHS Foundation Trust, Manchester Academic Health Sciences Centre (MAHSC), School of Biomedicine, University of Manchester, Manchester, UK M13 9WL

Simon Mitchell, Consultant Neonatologist, Newborn Intensive Care Unit, Central Mcr University Hospitals NHS Foundation Trust, Manchester, UK M13 9WL, College of Health & Social Care, University of Salford, Manchester, UK M6 6PU

Huma Aziz, User representative, Newborn Intensive Care Unit, Central Mcr University Hospitals NHS Foundation Trust, Manchester Academic Health Sciences Centre (MAHSC), Manchester, UK M13 9WL

Stephen A Roberts, Senior Lecturer in Medical Statistics, Health Sciences Methodology Research Group, University of Manchester, UK,

Tina Lavender, Professor of Midwifery, School of Nursing, Midwifery and Social work, University of Manchester, UK, M13 9PL
